# Acute myeloid leukemia following etoposide therapy for EBV-associated hemophagocytic lymphohistiocytosis: a case report and a brief review of the literature

**DOI:** 10.1186/s12887-016-0649-z

**Published:** 2016-07-29

**Authors:** Hua Pan, Dong-ning Feng, Liang Song, Li-rong Sun

**Affiliations:** 1 Department of Pediatric Hematology, Affiliated Hospital of Qingdao University, 16 Jiangsu Road, Qingdao, Shandong 266003 People’s Republic of China; 2 Department of Pediatrics, Qingdao Children’s Hospital, 6 Tong An Road, Qingdao, Shandong 266003 People’s Republic of China

**Keywords:** Etoposide, Hemophagocytosis, Secondary AML

## Abstract

**Background:**

Hemophagocytic lymphohistiocytosis (HLH) is a rare, life-threatening disorder characterized by prolonged fever, cytopenia, hepatosplenomegaly, rash, icterus, and other neurological symptoms. Successful treatment of HLH by etoposide has improved outcomes for children with HLH. However, the development of treatment-related acute myeloid leukemia (t-AML) after the usage of this drug is a concern.

**Case presentation:**

We report a case of acquired EBV-triggered HLH with progression to t-AML following etoposide therapy with cytogenetic abnormality for t (11; 19) (q23; p13) resulting in MLL gene fusion. The development of t-AML was detected 23 months after HLH diagnosis.

**Conclusions:**

Although the successful treatment of HLH by etoposide has improved outcomes for children with HLH, t-AML is a serious complication of topoisomerase II inhibitor therapy and is characterized by the presence of gene rearrangement. This study suggests that HLH patients undergoing therapy with HLH-2004 protocol need monitoring for future malignancy, especially in the initial 2 to 3 years.

## Background

Hemophagocytic lymphohistiocytosis (HLH) is a life-threatening disease of the immune system. HLH can be related into inherited gene defects or be triggered by severe infections, particularly with Epstein-Barr virus (EBV), rheumatoid disorders, or various malignant neoplasms. Familial hemophagocytic lymphohistiocytosis (FHL) is an immune disorder of autosomal recessive inheritance and is usually diagnosed within the first two years of life. Disease-causing mutations in gene PRF1, UNC13D, STX11, RAB27A, and STXBP2 (Munc18-2) have been found in FHL [1]. Hematopoietic stem cell transplantation (HSCT) is currently the only available curative method for FHL patients. Secondary HLH may occur anytime in life. EBV-associated hemophagocytic lymphohistiocytosis (EBV-HLH) syndrome is the most common trigger in the pediatric population. The mortality rate is 50 % if only supportive care is given for EBV-HLH patients [2]. HLH patients can die because of overwhelming infections or uncontrolled systemic inflammation and multi-organ failure. Therefore, HLH needs prompt diagnosis and immediate institution of therapy to improve the outcome in affected children. The control of the primary triggering factor and suppression of the overactive inflammation should be simultaneously pursued in treatment of HLH.

The successful treatment of HLH by etoposide has improved outcomes in children with HLH. However, development of t-AML related to the usage of this drug is a concern. In the present study, a case of EBV-HLH is reported with progression to t-AML following etoposide therapy with cytogenetic abnormality for t (11; 19) (q23; p13) resulting in MLL gene fusion.

## Case presentation

**Fig. 1:**
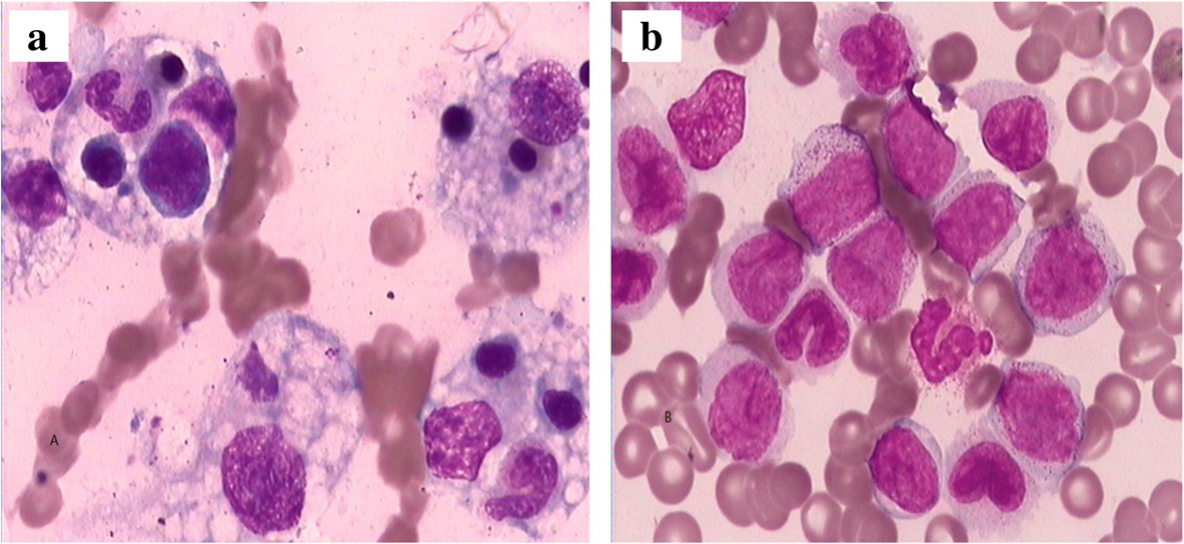
Bone marrow smears revealing hemophagocytic histocytes and numerous monoblasts and monocytes by light microscope. **a** Wright-Giemsa × 1000 (**b**) Wright-Giemsa × 1000

The patient was a 10-month-old Chinese female who was first diagnosed with HLH when she showed various symptoms, such as persistent high fever of unknown origin, seizures, jaundice, pancytopenia, coagulopathy, and hepatosplenomegaly. Physical examination also revealed lethargy, pallor, cervical lymph node swelling, and hepatosplenomegaly. Laboratory evaluations showed Hg b at 9.7 g/dl, H ct at 19.7 %, platelet at 60 K/μL, WBC at 2.67 K/μL, ferritin at 622 ng/mL, triglyceride at 532 mg/dl, ALT at 4068 U/L, AST at 9162 U/L, ALP at 1016 U/L, lactate dehydrogenase (LDH) at 12,108 U/L, total bilirubin at 100.9 umol/L, direct bilirubin at 51.5 umol/L, fibrinogen at 390 mg/dl, PT/PTT at 20.5/65.4 s, thrombin time of 46.7 s, and D-dimers at 851 ng/L. In summary, the patient fulfilled 5 out of 8 HLH-2004 criteria [3]. The cerebrospinal fluid examination showed protein at 3.5 g/L and pleocytosis at 20 × 10^9^/L. A magnetic resonance imaging (MRI) of the brain revealed no abnormality. Polymerase chain reaction (PCR) analysis for the EBV EBNA-1 was positive in peripheral blood. The EBV-DNA in blood was 6.25 × 10^4^ IU/mL. Bone marrow examination showed increased hemophagocytic histiocytes (HS) (Fig. 1a), which is consistent with HLH. Cytogenetic analysis was normal. A hereditary defect predisposing to HLH was not excluded. However, the long recurrence-free period argues against an hereditary background.

The patient received chemotherapy according to the HLH-2004 protocol, which includes etoposide, steroids, intrathecal methotrexate, and cyclosporin. The patient achieved complete remission of the disease at the end of the initial treatment period of 16 weeks. The patient did not achieved full remission of the disease until the end of the initial treatment period of 8 weeks. Treatment was thus continued until week 16 and was then stopped asl the parents declined further therapy. In total, the patient’s cumulative dose of etoposide was 2100 mg/m^2^ during the 16-week period.

After 23 months, the patient was readmitted with fever, leukocytosis, anemia, diffuse lymphadenopathy, and hepato-splenomegaly of 5 and 4 cm, which is palpable below the costal margins. LDH was elevated at 1788 U/L. Bone marrow examination revealed 89 % monoblasts, without evidence of HS (Fig. 1b). Flow cytometry studies indicated that 86.4 % of leukocytes were positive for CD33, CD7, CD11b, CD13, CD15, CD34, CD38, CD64, CD117, myeloperoxidase (MPO), and cytoplasmic CD68. Chromosome analysis of 48 h culture from bone marrow aspiration revealed complex karyotype aberrations. The karyotype was interpreted as 46, XX, d e r (11) t (11; 19) (q23; p13) inv (11) (q23 p15), and d e r (19) t (11; 19). The fusion gene was MLL/ELL. The diagnosis of AML (M5b) was made,. Induction chemotherapy with idarubicin and Ara-C (DA) was given, and the patient achieved complete remission in bone marrow morphology after two courses.

The patient is currently on chemotherapy for AML, and her bone marrow revealed no residual or recurrent leukemia cell after the initial complete remission.

## Discussion

We report a case of a 10-month-old Chinese female with EBV-associated HLH, who showed a progression to t-AML following etoposide therapy with cytogenetic abnormality for t (11; 19) (q23; p13) and MLL gene fusion, 23 months after the onset of HLH.

The t-AML following the etoposide therapy is not infrequent in malignant neoplasms such as leukemia and has first been reported in lung cancer in the 1980s [3]. About 2–12 % of patients treated with topoisomerase II inhibitors develop t-AML, frequently associated with translocations at 11q23 [4]. A review of related literature revealed 13 (11 secondary HLH and 2 FHL) cases of t-AML in HLH patients treated with etoposide (Table 1) [5–16].

**Table 1:** Previous reports of t-AML development in HLH patients treated with etoposide

Author and year of publication	Patient age (yr)/sex at onset of HLH	Disease as described	Etoposide cumulative doses prior to t-AML (mg/m2)	Additional anti-tumor agents for HLH	Time (mo) of t-AML from HLH therapy	Chromosome karyotype	The type of leukemia	Treatment for t-AML and outcome
Rama Chandran and Ariffin 2009 [7]	1.4/M	EBV-HLH	4800	Intrathecal MTX	36	t(15;17) (q22;q12)	M3	chemotherapy, alive
Kitazawa et al. 2001 [10]	4/F	EBV-HLH	3150	None	31	Normal	M2	Die after allo-HSCT
Takahashi et al. 1998 [11]	19/F	VAHS	900	Ara C	32	t(9;11) (p22;q23)	M4	Died after allo-HSCT
Rama Chandran and Ariffin 2011 [7]	1.5/M	VAHS	6150	Intrathecal MTX	48	t(15;17) (q21;q22)	M3	chemotherapy, alive
Rama Chandran and Ariffin 2011 [7]	3.5/F	VAHS	1200	none	24	Normal	M5	On chemotherapy, alive
Stine et al. 1997 [13]	11/M	VAHS	3100	Intrathecal MTX	26	t(9;11) (p22;q23)	M4	Died after allo-HSCT
Henter et al. 1993 [15]	3/M	FHL	20,500	Vm-26 3400 mg/m^2^, Intrathecal MTX	72	normal karyotype	MDS	Allo-HSCT, alive
Shamsian BS et al. 2010 [8]	12/F	HLH	400	MTX, prednisone, mercaptopurine	24	Normal	T-ALL	chemotherapy, die
Rudd et al. 2006 [16]	0.3/F	FHL	3150	None	24	t(15;17)	M3	chemotherapy, alive
Su et al. 2013 [5]	0.66/F	EBV-HLH	3520	None	18	t(15;17) (q22;q12)	M3	On chemotherapy, alive.
Seo et al. 2007 [9]	62/F	VAHS	300	None	31	Normal	M5	On chemotherapy, alive.
Ng et al. 2004 [12]	1.5/M	EBV-HLH	1350	None	6	t(9;11) (p22;q23)	AML	Allo-HSCT, alive
Ohtake et al. 2006 [14]	2/M	EBV-HLH	3900	None	14	t(9;11) (p22;q23)	AML	Allo-HSCT, alive

*FHL* familial hemophagocytic lymphohistiocytosis, *VAHS* virus related hemophagocytic lymphohistiocytosis, *allo-HSCT* allogenic hematopoietic stem cell transplantation

The aim of this paper is to fully describe the patient with an adverse effect of etoposide in the therapy of HLH and to characterize the risks and prognosis of t-AML.

HLH is a life-threatening hyperinflammatory disease caused by an uncontrolled and dysfunctional immune response of inherited or acquired immune deficiencies. All forms of HLH are characterized by an impaired function of natural killer (NK) cells and cytotoxic T-cells (CTL) [2]. EBV-associated HLH patients had mortality rates 14 times higher than when etoposide therapy was not initiated within the first four weeks [6]. The international protocol (HLH -94/2004), which consists of etoposide, dexamethasone, and cyclosporine, has been shown to induce remission of the disease. Etoposide is considered the most effective therapy for HLH, which acts through the inhibition of topoisomerase II and shows a high activity in monocytic and histiocytic disorders. The cumulative dose of etoposide of the HLH-2004 protocol 8 weeks induction is 1500 mg/m^2^. Imashuku et al. [17] reported that the chance of survival was significantly better for EBV-HLH patients receiving a cumulative dose of etoposide of 1000 to 3000 mg/m^2^ (*P* = .0001), compared with that of <1000 mg/m^2^ and without etoposide.

However, development of t-AML related to the usage of this drug is a concern. Most translocations of leukemia associated with epipodophyllotoxin disrupt an 8.3 kb breakpoint cluster region (b c r) between exons 5 and 11 of the MLL gene at chromosome band 11q23 [18]. Molecular characterization of breakpoints at 11q23 led to the identification of the MLL gene, which plays an important role in gene regulation during embryonic development and regulation of HOX genes during normal haematopoiesis [18]. MLL translocations are related to the occurrence of leukemia by gene fusion and resistance to stress-induced cell death and specific drugs. Four out of thirteen t-AML cases had MLL gene translocations at chromosome band 11q23.

Additional chromosome aberrations, such as t (15; 17), t (8; 21), inv (16), t (8; 16), t (11; 20), or t (11; 16), were also reported in younger patients and showed a shorter latency period between the primary tumor and t-AML [19]. Four of the 13 reported t-AML cases following etoposide in HLH therapy protocol were diagnosed with treatment-related APL with t (15; 17), as shown in Table 1.

Cases of t-AML following epipodophyllotoxin treatment generally occur within a relatively short latency period (ranging from 6 to 36 months), with 11 out of 13 cases developing t-AML within 36 months, as shown in these findings (Table 1). In the present study, the time interval between the occurrence of the hemophagocytic syndrome and the diagnosis of t-AML is 23 months.

One study demonstrates how early translocations first occur during the administration of topoisomerase II therapy. The MLL-GAS7 translocation was PCR-detectable by 1.5 months after starting treatment at low cumulative doses of the DNA topoisomerase II inhibitor doxorubicin [20]. Ng et al. [12] conservatively estimated that a 6.7 kb MLL cleavage fragment was detected in the bone marrow 3 months after a low dose of etoposide and 3 months prior to the diagnosis of overt t-AML. These studies may offer new leads for early detection and diagnosis as well as an understanding of the mechanism behind these translocations.

Different factors play a role for the risk of etoposide-related AML, which include the cumulative dose, schedule, other chemotherapeutic agents, and host factors. Imashuku et al. [17] investigated that the dosage of etoposide in the range of 1000–3000 mg/m^2^ was appropriate. However, three patients (shown in Table 1) who received less than 1000 mg/m^2^ cumulative dose of etoposide developed t-AML. The National Cancer Therapy Evalution Program (CTEP) showed that the calculated cumulative 6-year risks for the occurrence of t-AML for the low (<1.5 g/m^2^), moderate (1.5–2.99 g/m^2^), and high dose (≥3.0 g/m^2^) groups were 3.2, 0.7, and 2.2 %, respectively, and concluded that the incidence of t-AML after treatment with epipodophyllotoxins is not purely dose-dependent [21]. Winick [22] investigated the epipodophyllotoxin administration associated with the highest incidence (12 %) of t-AML in weekly or twice-weekly schedules in primary ALL. The administration of L-asparaginase, methotrexate, 6-mercaptopurine, cisplatin, and anthracyclines associated with epipodophyllotoxins is considered to increase the risk of t-AML [23]. Genetic polymorphisms in CYP3A5, CYP3A4, NQO1, and glutathione S-transferase genotypes have been demonstrated to play an important role in secondary malignancies [24, 25].

The outcome of t-AML is generally considered to be poorer than that of de novo AML. Remission is 80–90 % in t-AML, but long-term survival rates were 10–20 % [26]. Children with t-AML have a 5-year survival rate (23.7 %), which is significantly lower than that of children with AML as a first primary cancer (53.2 %) [26, 27]. Cytogenetics are important prognostic parameters in t-AML. Patients with t-AML who have favorable karyotypes, such as t (8; 21), inv (16), or t (15; 17), have good prognosis and can be given chemotherapyonly [27]. Su et al. [5] noted that all four t-APL patients receiving a combined application of all-trans retinoic acid and darubicin/Ara C had good diagnoses. Elliott et al. [28] observed that t-APL is sensitive to standard therapy. All four t-APL patients in Table 1 were alive after receiving chemotherapyonly. Schoch et al. [29] observed no statistically significant differences in overall survival (OS) of t-AML patientsin the group of the unfavorable and intermediate cytogenetics, whereas only the favorable subgroup showed a significantly shorter OS as compared with de novo AML. Outcomes were better among t-AML patients randomly assigned to receive intensively timed induction therapy than among those who received standard-timed induction [23]. Allogenic bone marrow transplantation was most effective for t-AML in remission at the time of transplant. Half (3/6) of the transplantation recipients for t-AML were alive, as shown in Table 1.

## Conclusions

In conclusion, therapy-related AML is the most serious complication of VP-16/etoposide-related chemotherapy for HLH.

## Abbreviations

CTEP, the national cancer therapy evalution program; CTL, cytotoxic t-cells; EBNA-1, Epstein-Barr virus nuclear antigen-1; HLH, hemophagocytic lymphohistiocytosis; HS, hemophagocytic histiocytes; HSCT, hematopoietic stem cell transplantation; MLL, mixed-lineage leukemia; MTX, methotrexate; NK, natural killer cells; OS, overall survival; PCR, polymerase chain reaction; T-AML, treatment-related acute myeloid leukemia
